# ACRODAT® and AcroVoice: an insight into a holistic approach to the management of acromegaly

**DOI:** 10.1007/s11102-019-00994-4

**Published:** 2019-10-09

**Authors:** Mark Lundie, Jill Sisco, Aart J. van der Lely

**Affiliations:** 1 Pfizer Canada, Kirkland, QC Canada; 2 Acromegaly Community, Grove, OK USA; 3 Erasmus University Medical Center, Rotterdam, The Netherlands

To the Editor,

We read the Letter to the Editor by Wang and Xing, 2019, entitled “AcroVoice: the controversial values in reflecting acromegaly disease activity” with some concern [1]. The authors misinterpreted the ACRODAT® study, and there is a need to correct this. Wang and Xing, 2019 presented a critique of the ACRODAT® study, not AcroVoice, and did not even reference the ACRODAT® paper [2] in their Letter to the Editor [1]. Moreover, Wang and Xing expressed concerns regarding the “rationality of the five parameters of ACRODAT®” [1].

As has been published, ACRODAT® was developed with expert opinion and validated in a rigorous study [2]. The resulting five dimensions (tumor status, insulin-like growth factor I [IGF-I] levels, comorbid conditions [diabetes, sleep apnea, and cardiac disease], symptoms, and quality of life [QoL]) offer a fast, simple tool to obtain an overview of patients’ disease and, when needed, help make informed treatment decisions [2]. The dimensions of ACRODAT® were carefully chosen to produce a balance between being practical to use and inclusive with respect to its power to reflect all of the important information on disease activity in acromegaly. Moreover, the dimensions of ACRODAT® are used by patients to gauge the severity of their disease, highlighting the importance of all components of acromegaly in the assessment of their condition. The inclusion of additional data or dimensions was evaluated by the expert panel; it was believed this did not add to the tool, and additionally, may confound potential issues in disease management.

With regard to the AcroVoice study, Wang and Xing raised concerns about the variability in patients’ responses to the discrete choice experiment (DCE) questions [1]. However, AcroVoice also employed a rigorous study design and the responses from the sample size of 100 patients exhibited a normal distribution, as would be expected from a large cohort, for which a pre-determined statistical analysis was employed to ascertain the robustness of the responses [3]. Moreover, Wang and Xing raised concerns that the patients’ responses or selections were too subjective since no specific values were used to define “slight” or “significant” abnormalities [1]. Again, the assumption is not accurate, and in fact, specific values were ascribed to slight or significant abnormalities for each parameter.

ACRODAT® and AcroVoice offer insights into a holistic approach to the management of acromegaly [2, 3]. Importantly, the AcroVoice study considered the patients’ perspectives surrounding their care and highlighted the strong desire of patients to have an active role in their disease management [3]. The AcroVoice study showed that patients with acromegaly valued both clinical factors and patient-centered factors. The study emphasized the importance of personalized care [3]. The AcroVoice study also showed that shared decision-making between patients and their doctors is important in making treatment decisions [3]. To support this conversation between patients and their healthcare providers, a Plain Language Summary (https://www.acromegalywest.com/blog) was developed.
